# Gendered perceptions of fairness in housework and shared expenses: Implications for relationship satisfaction and sex frequency

**DOI:** 10.1371/journal.pone.0214204

**Published:** 2019-03-20

**Authors:** Brian Joseph Gillespie, Gretchen Peterson, Janet Lever

**Affiliations:** 1 Department of Demography, University of Groningen, Groningen, The Netherlands; 2 Department of Sociology, University of Memphis, Memphis, Tennessee, United States of America; 3 Department of Sociology, California State University Los Angeles, Los Angeles, California, United States of America; London School of Hygiene and Tropical Medicine, UNITED KINGDOM

## Abstract

There is a demonstrated relationship between couples’ division of household chores—and, to a lesser extent, the division of shared expenses—and their relationship quality. Less is known, however, about whether and how individuals’ *perceived* fairness of these arrangements is associated with couples’ relationships in different ways. Using a gendered equity framework, and drawing on 10,236 responses collected via an online national news website, this study examines how equity evaluations of housework and shared expenses are related to relationship satisfaction and sex frequency among different-gender household partners. Consistent with previous findings, the results indicate that evaluations of unfairness to oneself are a stronger predictor of relationship quality than perceived unfairness to one’s partner. Additionally, fairness evaluations over shared expenses are a stronger predictor of relationship quality than perceived equity in housework. Incorporating notions about traditional gender norms and expectations into the justice framework, the results point to some variation in relationship outcomes based on men’s and women’s differential equity evaluations.

## Introduction

An important component of married and cohabiting unions is how housework and shared expenses are divided. These arrangements often warrant careful negotiation that requires trust and touches on issues related to equality, gender, and social rules and expectations [1,2]. For this reason, the divisions of household labor and shared expenses have been of interest to family economists, sociologists, and psychologists for decades (see [3,4]). Across a wide range of cultural contexts, research typically indicates that women shoulder a greater share of the housework load, often approaching a 2:1 ratio [5, 6, 7]. The division of shared expenses by cohabiting and married couples also is associated with relationship dynamics and evaluations of satisfaction with one’s relationship [8], especially because decisions about the division of housework and shared expenses are interrelated [9].

A great deal of research on household inequality focuses on *relative contributions* to housework (e.g., [10]); however, the *perceived fairness* of these arrangements is often considered more important than the proportionality of individuals’ contributions [11, 12, 13]. In fact, a recent finding that housework arrangements help predict sexual satisfaction was attributed, in part, to “the increasing role of perceived equity as a mechanism linking the division of housework to sex” [14].

The management of shared finances, which involves individual and common interests, also requires careful negotiation. Like housework, these negotiations can lead to assessments of fairness that influence relationship quality. However, this topic has received less attention than housework, despite a strong association between financial issues and relationship outcomes [4, 15, 16]. An even smaller literature has explored how perceived fairness in couples’ economic arrangements might increase relationship satisfaction (e.g., [17, 18, 19]).

The current study unites research on the division of household labor and the division of shared expenses to understand how individuals’ equity evaluations are associated with their perceptions of relationship quality. In so doing, we simultaneously examine individuals’ reports of perceived fairness in the division of household labor and shared expenses. Combining these two fairness processes in one study allows for a more complete examination of household management and relationship dynamics. The focus on *perceived* fairness distinguishes this research from some previous work as it does not assume any particular arrangement would be unfair (e.g., one person doing most of the housework). Rather, individuals make their own assessments about the fairness of household arrangements for themselves (ego equity evaluations) and for others—in this case, their romantic partners.

Gender ideologies may influence how actors interpret an objective inequality in household management [1]; therefore, assessing *perceived* fairness can be especially useful in understanding different-gender relationship outcomes. Since fairness assessments may at least partially hinge upon traditional gender norms and expectations regarding both housework and finances, another goal of this study is to examine how *gendered* equity evaluations of household arrangements are linked to relationship satisfaction and sex frequency.

## Prior research and theory

Recent research indicates that the housework gender gap might slowly be closing [5, 6, 20, 21]. Perhaps somewhat counter-intuitively, there is some evidence that this cultural shift has led to greater happiness and improved work-life balance for men who do more housework, but less so for wives whose husbands do more housework [6, 22, 23]. This gender difference might be due to the fact that women still shoulder the greater burden of household labor even when their partners do more [24]. Moreover, women often feel more responsible for maintaining the household [25], which may increase the amount of time not only doing the work but also thinking about the work, even when some tasks are delegated to others [26]. As such, women may be less satisfied with household arrangements, in part, due to their continuing responsibility for managing and monitoring who does what in the household.

Regarding couples’ income organization, money management strategies have been distinguished based on whether they are pooled, partially joint, or separate [27]. Like housework, these strategies can lead to assessments of fairness that influence perceptions of relationship quality. However, the *actual* arrangement that couples use (i.e., separate purses, pooled, partially joint) is less central for the purposes of this study than individuals’ perceived fairness of said arrangement.

There is also reason to believe that household arrangements are associated with relationship outcomes, especially sexual satisfaction and the frequency of sexual activity. However, results have been mixed (see [28, 29]), possibly due to generational differences in the data used [14]. To build on these past studies, we examine the association between perceived fairness in household arrangements and two mutually-reinforcing measures of relationship quality: sex frequency—the strongest perceptual/behavioral measure linked to sexual satisfaction [30, 31, 32]—and relationship satisfaction.

### Theoretical framework

We draw on notions of equity in the justice framework of social psychology [33, 34]. Equity theory posits that individuals are motivated by evaluations of fairness in their interactions. Perceived equity or inequity regarding inputs and yields will impact the relationship [33]. More specifically, this framework proposes that individuals who perceive any *inequity* in their interpersonal interactions will experience negative outcomes. These outcomes are based on perceived inequity for oneself and—somewhat less strongly—perceived inequity for one’s partner [35].

In order for a particular arrangement to be perceived as fair, individuals do not need to contribute equally. The ratio between perceived inputs and yields are what drives equity evaluations (e.g., sharing finances proportionally rather than equally). Early research by Adams [33] and Homans [36] showed how transactional relationships can be harmed when there is inequity—underreward in a transaction leads to feelings of distress while overreward can lead to feelings of guilt. As such, we expect any perceived unfairness in the division of housework and shared finances (whether unfair to oneself or to one’s partner) to result in perceptions of lower relationship quality than if the household arrangement is perceived as fair.

Assessments about equity in household arrangements depend on two concurrent processes. Individuals assess their own contributions relative to their partner’s inputs. They also evaluate whether the arrangements are reasonably fair, unfair *to oneself* (ego unfairness), or *unfair to one’s partner* (unfairness to other). Evaluations of ego unfairness (for oneself) is presumed to be qualitatively different from perceived unfairness for others in terms of emotional reactions [35] and sensitivity to what constitutes unfairness [37, 38]. For example, those who perceive arrangements to be ego unfair will critique their marriage more harshly than if the arrangement is unfair to their partner [39, 2, 12]. Therefore, another objective of this study is to examine how differential evaluations of ego vs. partner unfairness are associated with relationship outcomes.

Specifically, we predict the following:

Hypothesis 1: When compared with household arrangements that are perceived as "mostly fair," perceived unfairness in the division of housework and shared finances (whether unfair to oneself or to one’s partner) will be *negatively* associated with relationship quality.

*Hypothesis 1*.*1*: When compared with individuals who report that their household arrangements are “mostly fair,” we expect a stronger negative relationship between ego unfairness and relationship quality than for unfairness to partner.

Building on findings that “financial disagreements are stronger predictors of divorce relative to other common marital disagreements” [16], we also predict:

*Hypothesis 1*.*2*: Evaluations of equity in the division of shared expenses will be a stronger positive predictor of relationship quality than evaluations of equity in housework.

### Gendered equity assessments

Much of the literature on housework, income provision, and relationship quality focuses on the power and importance of gender norms for individual behavior within different-gender intimate relationships (e.g., [40, 41, 9]). Viewing the equity framework through a gendered lens would suggest that the values placed on different inputs and outcomes differ between men and women, leading to different gendered assessments of the relationship. For example, Kroska [42] demonstrates that affective meanings tied to household tasks are not simply gendered, but may reflect patterns in paid and unpaid work and notions of housework as obligation for women versus choice for men.

Adopting a gendered framework allows us to assess whether men and women are invoking traditional or egalitarian ideologies in their equity evaluations. Recent research has argued that changes in the home and economic spheres are driving ideological shifts toward egalitarianism [14, 43]. Insofar as these shifts have occurred—and traditional notions of gender are losing their cache—individuals’ gendered perceptions of fairness in housework and finances would reflect such a shift. While gender ideologies have long been used as an underlying explanation for differences between objective and subjective fairness in divisions of household labor (e.g., [44, 45]), the recent work on ideological shifts and the affective meanings of household management points to other factors connected to gender ideologies that may be at play. Some of Kroska’s [42] findings indicate that women may feel more accountable for household labor whereas men view their contributions as a choice, leading women to feel more negatively about disproportionate burdens of housework while men feel more positively about making contributions to the household.

The current study incorporates measures for hours of paid work and accounts for time spent commuting to paid work in individuals’ assessments of fairness, which seem on the surface to be economic variables; however, just under the surface, they too are gendered in the sense that some men assume greater responsibilities for paid work, seeing breadwinning as their primary role. Men may be willing to commute further not only to maximize economic reward but also in deference to their wives taking great responsibility for child-rearing and choosing work closer to home to spend more time with the children [46].

In other words, individuals’ equity evaluations can be rooted in traditional notions of gender simply because normative conceptions of gender are so deeply embedded. Traditional scripts provide a rigid definition of men’s and women’s gendered roles and expectations [47]. Their embeddedness is reinforced through gender display or “doing gender” out of concern for mischaracterization or being held accountable for going off-book [40].

A primary goal of this study is to examine whether men and women invoke *gendered* equity evaluations and, if so, how their assessments are linked to their relationship satisfaction and sex frequency. We examine individuals’ perceptions of fairness for two historically gender-stereotyped arrangements in relationships, the division of housework and the division of shared expenses. Income provision has traditionally been the realm where men display gender as providers—a cultural reflection of the male domination of financial resources [9, 48]. At the same time, housework is tied to traditional notions of woman as caretaker, reflecting women’s restriction to the domestic sphere [49, 50]. Given the gendered notions tied to each of these inputs into a relationship, perceptions of fairness in each of these spheres would also be gendered. Equity theory ties the fairness of inputs to the fairness of outcomes so gendered evaluations of inputs are then argued to lead to gendered evaluations of outcomes.

Specifically, drawing on a gendered interpretation of the equity framework, we predict the following:

Hypothesis 2: We expect the association between fairness assessments in housework and shared income on relationship quality to differ for men and women.

*Hypothesis 2*.*1*: When compared with housework arrangements perceived as mostly fair or unfair to their partner, we expect men who perceive the division of housework as ego unfair to report lower relationship quality than women who perceive the division of housework as ego unfair.
*Hypothesis 2*.*2*: When compared with financial arrangements perceived as mostly fair or unfair to their partner, we expect women who perceive the division of shared expenses as ego unfair to report lower relationship quality than men who perceive the division of shared expenses as ego unfair.

## Method

### Data

Data for this project come from the “Money, Sex & Love Survey” which was posted on NBCNews.com for ten days in 2008. An invitation to participate in a survey appeared on the front page of the Financial News section and periodically on the website’s main homepage. Respondents were provided with an opportunity to view the privacy agreement and asked for their birth year; those under age 18 were dismissed as too young to participate. To prevent the same individual from responding to the survey more than once, a software program denied multiple responses from any given computer.

NBCNews.com (formerly known as msnbc.com) remains one of the most popular news sites in the USA; Nielsen/Net Ratings in May 2009 showed it had over 37 million unique users within the US, giving it the number one ranking among global news sites, around the time the survey was posted. The broad-based appeal of the website provided a diverse national sample and an opportunity to compare men and women who differed substantially in their perceptions about housework and shared income.

As consultants on this survey, as well as on previous ones posted on the business or health sections of this news website, we had delayed access to anonymized data sets for the sole purpose of scientific re-analyses. Results have been published in dozens of peer-reviewed journals, adding to knowledge on a variety of topics, including attitudes towards female bosses [51], close adult friendship [52], predictors of sexual activity in long term couples [53], the use of online dating sites [54], infidelity [55], body image [56], and paying for dates [57]. Data for the current study are available as a Supplemental Information file that accompanies this manuscript [S1 Dataset].

### Sample

Because we were interested in how traditional versus emerging gender norms impacted evaluation of equity or non-equity in relationships, we restricted our sample to respondents in different-gender couples who live together. We further restricted our sample to those who answered *both* items related to fairness in the division of housework and shared finances. Individuals who reported being gay/lesbian or bisexual (*n* = 569) were removed. The final sample (*N* = 10,236) was 65% male, 89% married or remarried, and had an average age of 42.6 (*SD* = 11.8). Additional information about sample characteristics is presented separately for men and women in Table 1.

**Table 1:** Sample characteristics (*N* = 10,236).

	Men (*n* = 6,637)		Women (*n* = 3,599)	
	N (%)	Mean (SD)	N (%)	Mean (SD)
**Dependent Variables**				
Relationship Satisfaction (Range: 1–7)		5.3 (1.8)		5.5 (1.8)
Sex Frequency (Range: 0–8)		4.2 (1.7)		4.6 (1.7)
**Independent Variables**				
*Fairness of Housework Division*				
Unfair to Partner	1,927 (29.0)		310 (8.6)	
Mostly Fair	3,667 (55.3)		1,780 (49.5)	
Ego Unfairness	1,043 (15.7)		1,509 (41.9)	
*Fairness of Shared Expenses*				
Unfair to Partner	175 (2.6)		345 (9.6)	
Mostly Fair	5,398 (81.3)		2,691 (74.8)	
Ego Unfairness	1,064 (16.0)		563 (15.6)	
**Demographic Controls**				
Married	6,220 (93.7)		2,909 (80.8)	
Children	4,377 (66.0)		1,985 (55.2)	
Age		45.2 (11.6)		37.8 (10.8)
Relationship Length in Years		12.2 (7.7)		8.0 (7.6)
Hours Worked		44.0 (15.8)		36.3 (17.1)
Household Income (Thousands)		109.9 (141.5)		52.7 (76.6)
*Highest Degree*				
High School or Less	455 (6.9)		328 (9.1)	
Some College/A.A.	1,956 (29.5)		1,315 (36.5)	
College Degree	2,633 (39.7)		1,342 (37.3)	
Graduate Degree	1,593 (24.0)		614 (17.1)	

### Measures

#### Dependent variables

*Relationship satisfaction* is based on the respondent’s reported level of satisfaction with their current relationship ranging from: (1) *very dissatisfied* to (7) *very satisfied*, with (4) as a neutral midpoint. A second dependent variable, *sex frequency*, is based on an item asking the respondent to report how frequently they have sex with their partner. The response options were (0) *not at all*, (1) *once a year*, (2) once every few months, (3) *once a month*, (4) *two or three times a month*, (5) *once or twice a week*, (6) *three to four times a week*, (7) *five or more times a week*, and (8) *more than once a day*.

#### Independent variables

To assess *perceived fairness of the division of housework*, respondents were asked: “Considering work/commute schedules, would you say the division of your household/childcare chores is fair?” The response categories included: “no, I do way too much of the work” and “no, I do somewhat more than I should” (coded as “perceived ego unfairness”); “no, my partner does way too much of the work” and “no, my partner does somewhat more than he/she should” (coded as “perceived unfairness for partner”); and “yes, it’s fair enough” (coded as “mostly fair”). Sensitivity analyses using the full measure of perceived fairness in housework produced virtually the same results as those using the collapsed, three-point measure so we elected to retain the three-point measure for ease of interpretation.

To assess the *fairness of the division of household expenses*, respondents were asked: “In your relationship, is the way you handle your shared expenses fair?” Response categories included: “I pay more than I should” (coded as “perceived ego unfairness”); “it’s about right” (coded as “mostly fair”); and, “my partner pays more than she/he should” (coded as “perceived unfairness for partner”).

#### Covariates

A variable for *gender* indicated whether the respondent was (1) female or (0) male. A dichotomous variable for *marital status* indicated whether the respondent was (1) married or (0) in another arrangement (i.e., never married or divorced) yet cohabiting. A dichotomous measure indicated whether the couple had any financially-dependent children or stepchildren, coded 1, else = 0. Continuous variables marked the respondent’s *age* and number of *years in their current relationship*.

Participants indicated their *hours of paid work* per week. Ordered response options were coded at the midpoint: (2) *1–4 hours*, (10) *4–14 hours*, (20), *15–24 hours*, (30) *25–34 hours*, (40) *35–44 hours*, (50) *45–54 hours*, (60) *55–64 hours*, (70) 65–74 hours, (80) *75–84 hours*, and (90) *85+ hours per week* and (0) classified *unemployed* individuals and those not in the labor force. Respondents reported the range of their *personal annual income*. For missing responses on this variable (*n* = 336), we imputed the median response ($65,000). To facilitate interpretation of regression coefficients, income was divided by 1,000. Lastly, an ordinal measure for *highest degree completed* identified whether respondents had obtained a *high school education or less*, *some college* or A.A., a *college degree*, or a *graduate degree*.

#### Analytic strategy

Tables 2 and 3 present differences between men and women in terms of relationship satisfaction and sex frequency by fairness assessments of housework and shared expenses. In order to examine the relative contribution of equity evaluations for housework compared with shared expenses, the first series of multivariate models (Table 4) are based on OLS regressions with standardized regression coefficients (β). In Table 4, the reference category for the equity evaluation measures was that the arrangement was “mostly fair.”

**Table 2:** Relationship satisfaction by gendered equity evaluations (*N* = 10,236).

Perceived Fairness	Housework				Shared Expenses			
	*Men*	*Women*	*t (df)*	*d*	*Men*	*Women*	*t (df)*	*d*
Unfair to Partner	5.4 (1.7)	5.7 (1.5)	-2.7 (2,235)**	-.17	5.0 (2.0)	5.6 (1.8)	-3.1 (518) **	-.29
Mostly Fair	5.4 (1.7)	5.9 (1.6)	-9.0 (5,445)***	-.26	5.5 (1.6)	5.7 (1.6)	-4.6 (8,087)***	-.11
Ego Unfairness	4.3 (1.9)	4.9 (1.9)	-7.6 (2,550)***	-.31	4.1 (1.9)	4.3 (2.0)	-2.6 (1,625)**	-.14

** *p* < .01

*** *p* < .001.

**Table 3:** Sex frequency by gendered equity evaluations (*N* = 10,236).

Perceived Fairness	Housework				Shared Expenses			
	*Men*	*Women*	*t (df)*	*d*	*Men*	*Women*	*t (df)*	*d*
Unfair to Partner	4.3 (1.6)	4.5 (1.7)	-2.6 (2,235)**	-.16	4.1 (1.9)	4.9 (1.6)	-5.1 (518)***	-.47
Mostly Fair	4.3 (1.8)	4.9 (1.7)	-12.1 (5,445)***	-.35	4.3 (1.7)	4.7 (1.7)	-10.7 (8,087)***	-.25
Ego Unfairness	3.7 (1.8)	4.4 (1.8)	-9.4 (2,550)***	-.38	3.7 (2.0)	4.3 (2.0)	-5.5 (1,625)***	-.29

** *p* < .01

*** *p* < .001

**Table 4:** OLS regression of equity evaluations and relationship outcomes.

*N* = 10,236	Relationship Satisfaction		Sex Frequency	
	Model 1.1	Model 1.2	Model 2.1	Model 2.2
**Independent Variables**				
*Fairness of Housework Division*				
Ego Unfairness	-0.16***	-0.18***	-0.06***	-0.10***
Mostly Fair (Reference)				
Unfair to Partner	-0.03***	-0.00	-0.04***	-0.01
*Fairness of Shared Expenses*				
Ego Unfairness	-0.25***	-0.26***	-0.09***	-0.12***
Mostly Fair (Reference)				
Unfair to Partner	-0.02	-0.05***	0.03**	-0.02*
**Demographic Controls**				
Female		0.05***		0.03**
Married		-0.04***		-0.12***
Children		-0.07***		0.07***
Age		-0.00		-0.16***
Relationship Length		-0.16***		-0.28***
Hours Worked		-0.01		0.00
Personal Income (Thousands)		-0.00		0.05***
*Highest Degree (Reference*: *College)*				
High School or Less		-0.00		0.02*
Some College or Associate's Degree		-0.02*		0.03***
Graduate Degree		-0.01		0.01
Model Fit (Adjusted R^2^)	.11	.16	.01	.22

**Table Note**: Standardized Coefficients (β)

* *p* .05

** *p* < .01

*** *p* < .001.

Given that both dependent variables (relationship satisfaction and sex frequency) and the main independent variables (equity evaluations of the division of housework and shared expenses) describe overlapping characteristics in a relationship, subsequent analyses in Table 5 are based on seemingly unrelated regression models (SUR) with unstandardized regression coefficients (*b*). This approach is advantageous for family research where it is unlikely the dependent variables are independent (e.g., [58]). SUR appropriates for this by accounting for the correlation in the error terms in two linear equations and estimates the parameters of the equations jointly [59, 60].

**Table 5:** Seemingly unrelated regression (SUR) of equity evaluations and relationship outcomes.

*N* = 10,236	Relationship Satisfaction	Sex Frequency
**Independent Variables**		
*Fairness of Housework Division*		
Ego Unfairness	-0.78***	-0.49***
Mostly Fair (Reference)		
Unfair to Partner	0.02	-0.01
*Fairness of Shared Expenses*		
Ego Unfairness	-1.28***	-0.59***
Mostly Fair (Reference)		
Unfair to Partner	-0.59***	-0.34**
**Interactions**		
*Housework and Gender*		
Ego Unfairness x Female	0.07	0.18*
Unfair to Partner x Female	-0.14	-0.32**
*Shared Expenses and Gender*		
Ego Unfairness x Female	0.04	0.09
Unfair to Partner x Female	0.33*	0.27
**Controls**		
Female	0.17***	0.07
Married	-0.22***	-0.66***
Children	-0.27***	0.26***
Age	0.00	-0.02
Relationship Length	-0.04***	-0.06***
Hours Worked	-0.00	0.00
Household Income	0.00	0.00***
*Highest Degree (Reference*: *College)*		
High School or Less	-0.03	0.14*
Some College or Associate's Degree	-0.08*	0.12***
Graduate Degree	-0.05	0.02
Constant	6.5***	6.5***
Model Fit	.16	.22

**Table Note**: Unstandardized Coefficients (β)

* *p* .05

** *p* < .01

*** *p* < .001.

## Results

The average relationship satisfaction score was 5.3 (SD = 1.8) and sex frequency was 4.3 (SD = 1.8). A majority of the sample reported fairness in shared expenses and housework; 79% reported the division of shared expenses as fair, while 53.2% reported fairness in housework. Nearly 25% of respondents perceived ego unfairness in housework and 15.9% reported ego unfairness in shared expenses.

### Bivariate analyses

Preliminary analyses (not shown) indicated that men reported significantly lower relationship satisfaction scores (M = 5.3, SD = 1.8) than women (M = 5.5, SD = 1.8) (*t* = -5.4, df = 10234, *p* < .001, *d* = -.11). Men also reported significantly lower sex frequency (M = 4.2, SD = 1.7) than women (M = 4.6, SD = 1.7) (*t* = -13.1, df = 10234, *p* < .001, *d* = -.27). Additionally, there was a statistically significant relationship between individuals’ evaluations of housework fairness and their evaluations of the fairness of shared expenses (χ^2^ = 524.9, *p* < .001).

Tables 2 and 3 present differences in mean relationship satisfaction between men and women across different equity evaluations for housework and shared expenses. To correct alpha for the analysis of multiple subgroups, only results significant at the .01 level or lower are reported and discussed [61]. Since our large sample size provides the power to detect even small associations in analyses conducted with the overall sample, we highlight the effect size Cohen’s d for comparing means, which assesses the size of the difference between two means in standard deviation units.

Cohen [62] suggested that, in general, d values of .20, .50, and .80 be considered small, moderate, or large, respectively. To indicate the direction of the gender difference, negative *d* values indicated that women had higher relationship quality. For example, *d* = -.17 in Table 2 points to a small but significant difference—when housework was ego unfair, women reported higher relationship satisfaction than their male counterparts (*p* < .001).

Consistent with the bivariate measures of relationship quality discussed above, men reported significantly lower relationship satisfaction and sex frequency than women across all contexts of perceived fairness (all *p*s < .01). For both dependent variables, the difference between men and women was especially pronounced when (a) the division of shared expenses was perceived as unfair to partner and (b) housework arrangements were perceived as ego unfair. While this does not provide a clear assessment of gender differences in equity evaluations for the two outcomes, the results do provide preliminary support for Hypothesis 1—perceptions of unfairness (i.e., for self or other) are associated with lower relationship quality. These results also provide some preliminary support for Hypothesis 2—the effect of perceived fairness in housework and income on relationship quality differs between men and women.

### Multivariate analyses

#### Individual OLS regressions

Table 4 presents the results of separate OLS regressions for each dependent variable. In order to assess the relative impact of housework evaluations versus income sharing evaluations on the dependent variables, standardized coefficients are reported and discussed. For all multivariate analyses, variance inflation factors indicated there was no severe multicollinearity in the models (average VIF = 1.3). Analysis of the correlation matrix (not shown) indicated that none of the observed relationships between the independent variables in the models were very strong; the strongest correlation (.61) was between age and relationship length. The results for housework and income sharing in OLS models run individually for men and women (not shown) did not differ substantially from the full model discussed in the text and presented in Table 4. These auxiliary results are available upon request.

Hypothesis 1: Perceived fairness and relationship quality. Baseline models (Models 1.1 and 2.1) indicated that perceived unfairness for both self and partner were associated with significantly lower relationship satisfaction and sex frequency (*p* < .01) than perceived fairness, with the exception of perceived unfairness for partner in shared expenses. However, in Models 2.1 and 2.2, which included covariates, the effect of unfairness to partner was diminished for household chores but significant and negative for shared expenses (*p*s < .001). This suggests that different mechanisms are at play for assessments of the fairness of shared expenses and for housework. As such, there was only partial support for Hypothesis 1, that any perceived unfairness (for self or partner) would be negatively associated with relationship outcomes. This expectation was borne out for ego evaluations and perceived unfairness to partner in shared expenses. However, assessments of unfairness to partner in housework was not associated with relationship satisfaction or sex frequency when compared with those who perceived the arrangements as “mostly fair.”

Hypothesis 1.1: Perceived ego unfairness versus perceived partner unfairness. Overall, the results in Table 4 supported *Hypothesis 1*.*1*—that perceived ego unfairness would be a stronger predictor of relationship quality than perceived partner unfairness. In each of the OLS regression models, the standardized coefficients indicated that ego unfairness was a stronger predictor of relationship quality than partner unfairness. This finding supports justice research that demonstrates stronger effects of underreward on people’s perceptions (distress) compared to overreward (guilt).

Hypothesis 1.2: Evaluations of shared expenses versus evaluations of housework equity. Additionally, the results support *Hypothesis 1*.*2*—that perceived fairness about shared expenses would be a stronger predictor of relationship quality than perceived fairness about housework. Based on the standardized coefficients in Model 1.2, perceived ego unfairness in the division of shared expenses was the strongest predictor of relationship satisfaction (β = -.26, *p* < .001). It was also a strong predictor of sex frequency (β = -.12, *p* < .001), following only relationship length in relative importance. As such, perceived fairness regarding shared finances was a stronger predictor of relationship quality than perceived fairness in the division of housework.

#### Seemingly unrelated regression

The zero-order correlation between the residuals for each model is 0.4 (*p* < .001). Results from the Breusch-Pagan test of independence showed that the two dependent variables were not statistically independent [χ^2^(1) = 1518.1, *p* < .001], indicating a significant correlation in the error term in the model for relationship satisfaction and that for sex frequency. Accordingly, SUR is an improvement upon individual OLS models [63].

Table 5 presents the results of two SUR models run simultaneously on each dependent variable. Providing additional support for Hypothesis 1.1, the results of the SUR indicate that housework and financial arrangements perceived as ego unfair were associated with significantly lower relationship satisfaction and sex frequency when compared with mostly fair arrangements (all *p*s < .001).

Hypothesis 2.1: Lower relationship quality for men who perceive housework arrangements as ego unfair than women who perceive the division of housework as ego unfair. To test the hypotheses for specific gendered arrangements, interaction terms were included in Table 5. Regarding gendered equity evaluations, we first expected that men would report lower relationship quality if they perceived housework arrangements as ego unfair compared with women who perceived housework as ego unfair. This hypothesis was not supported by the results. The interaction term for perceived fairness in housework and gender is significant; however, the relationship differs based on individuals’ perception of unfairness as ego or partner and relationship outcome.

To facilitate interpretation of the interaction terms, Table 6 presents estimated cell means for the interactions between gender and perceived fairness in housework and shared expenses. Pairwise tests for differences in the estimated marginal means point to two significant relationships between gender and perceived fairness in housework. First, women who reported that housework was unfair to her partner reported significantly higher relationship satisfaction than men who reported that housework was unfair to his partner (p < .001). At the same time, women who reported that housework was unfair to her partner reported less frequent sex than men who reported housework was unfair to his partner (p < .001). All other pairwise tests were nonsignificant.

**Table 6:** Adjusted cell means for interaction terms.

	Relationship Satisfaction	Sex Frequency
**Perceived Housework Fairness**		
Unfair to Partner x Male	5.06	4.21
Mostly Fair x Male	5.05	4.22
Ego Unfair x Male	4.27	3.73
Unfair to Partner x Female	5.21	4.08
Mostly Fair x Female	5.34	4.41
Ego Unfair x Female	4.63	4.09
**Perceived Fairness of Shared Expenses**		
Unfair to Partner * Male	4.83	4.02
Mostly Fair x Male	5.42	4.36
Ego Unfair x Male	4.13	3.77
Unfair to Partner x Female	5.30	4.31
Mostly Fair x Female	5.56	4.39
Ego Unfair x Female	4.31	3.88

Hypothesis 2.2: Lower relationship quality for women who perceive the division of shared expenses as ego unfair than men who perceive financial arrangements as ego unfair. The results did not support the hypothesis that women who report ego unfairness in shared expenses would report lower relationship quality than men who perceived shared expenses as ego unfair. The interaction term between gender and the perceived fairness of shared expenses was not significantly associated with either relationship outcome (Models 4.1 and 4.2).

## Discussion

This study brought together the literatures on household labor and household expenses to examine how equity evaluations are associated with relationship outcomes. Since fairness assessments are often made within the context of traditional gender norms regarding housework and finances, we also examined associations between *gendered* equity evaluations and relationship outcomes. Integrating ideas about equity from the justice literature into analyses of household arrangements allows for the elaboration of both lines of inquiry. Justice researchers benefit from the application of the theoretical concepts to situations outside the laboratory, and family researchers benefit from a broadening of the theoretical orientations used in their work.

One key feature of our study was the simultaneous examination of perceived equity of household labor and shared expenses. Many studies focus exclusively on inequality in housework [50, 64, 41, 65] or other household activities [66, 67, 68, 69]. Bringing together considerations of the perceived fairness of the divisions of labor and of expenses provides a more complete picture of the relationship context within which outcomes such as sexual frequency and relationship satisfaction are manifest.

Building on this strength, this study examined two dependent variables that are connected to relationship quality—and quality of life, overall—an emotional outcome (relationship satisfaction) and a behavioral outcome (frequency of sexual activity). Simultaneous equation modeling with SUR allowed us to examine whether and how different fairness assessments in household arrangements were linked to these two outcomes.

Another way this study builds upon prior work is that the phrasing of the item assessing perceptions of fairness in housework departed from traditional operationalizations of the concept. Others have adopted a broader approach. For example, Lively et al. [2] used “How fair do you feel the division of work around the house is in your household? Would you say it is fair to both you and your spouse or partner, unfair to you, or unfair to your spouse or partner?” Our question was strategically designed to account for both work schedule and commute time as work-related factors that likely influence how much household labor a person does.

Whereas some might argue that asking respondents to consider commute time might prime the respondent to give her or his partner an excuse for not doing more—that is, commute differences get woven into “family myths” in the way Hochschild [50] uses the concept to justify inequalities—we see it as a reasonable economic variable that has been largely ignored. There is evidence that men spend considerably more time commuting to work [46], and it is reasonable to view couples as rational partners who consider disproportionate time inputs to family economy.

Our findings contribute to research using the justice framework to examine how couples’ intimate relationships relate to their perceived fairness in household arrangements. First, we found that overall assessments of unfairness for both oneself or one’s partner led to declines in relationship quality—but this relationship held only for shared finances, not housework, in the full OLS models. Thus, we found partial support for equity theory’s premise that underreward and overreward can lead to feelings of distress and guilt respectively [33]. The reason that perception of unfairness for one’s partner in housework is not significantly associated with relationship factors might be related to another main finding of this study—that perceptions of fairness in the division of shared expenses is a stronger predictor of relationship quality than are perceptions of fairness in housework.

It might be that perceived inequities in housework can be remedied more easily than financial arrangements (e.g., housework can be reallocated or outsourced to establish equilibrium in times of need). On the other hand, financial matters are less flexible and require careful negotiation over finite resources, leading to feelings of guilt when the scales of justice are tilted in one’s own favor. An additional explanation might be that financial inequities are more salient because they are rooted in discrete negotiated transactions whereas housework inequity only becomes apparent over time [70].

We also found that perceived ego inequities are stronger predictors of relationship quality than evaluations of inequity for one’s partner. Further, in light of our incorporation of different gender-stereotyped household arrangements and outcomes, we show that these assessments are experienced across multiple relationship domains. While these findings seem less novel than the others, they do help substantiate recent literature speculating that evaluations of ego unfairness are qualitatively different from perceived unfairness for others [35]. One explanation for the differential magnitude of these fairness assessments might stem from attribution theory [71].

This perspective suggests that individuals, when accounting for their own actions, will recognize and attribute structural and situational explanations for their behaviors. On the other hand, when making assessment about *others’* actions, individuals tend to attribute personal explanations and fail to recognize situational factors. In this sense, when an individual perceives that the division of housework is unfair to their partners, their assessments are more likely to include their own additional compensatory inputs to the relationship, thereby absolving them of feeling guilt over an otherwise unfair housework arrangement. Along the same lines, when individuals feel like household arrangements are unfair to themselves, they might fail to account for their partner’s contributions to the relationship, leading to stronger emotional responses.

Building on the first series of hypotheses regarding fairness assessments and household arrangements, a second series of hypotheses was based on gendered equity evaluations. These findings indicated that gender differences in fairness assessments do, to some extent, exist. However, equity evaluation processes operated differently depending on the household arrangement domain, perceived fairness for ego or partner, and the outcome variable.

Notably, women who perceived that income sharing was unfair to their partners reported higher relationship satisfaction than men who perceived that income sharing was unfair to their partners. Apart from these findings, there was little overall support for our proposed gendered equity framework—relationship quality is indeed conditioned by gendered evaluations of fairness but in seemingly more complicated ways.

There are several plausible explanations for why our gendered equity evaluation hypotheses were not entirely borne out for relationship quality. First, experimental research suggests that men more commonly make financial assessments based on equity, while women tend to make assessments based on equality [72]. These distinctions likely relate to what individuals perceived as fair or unfair within their union. Since our argument was based on equity theory, it may be that the use of different fairness standards by men and women meant that the connection between the gendered evaluations of the fairness of inputs was not as closely connected to relationship outcomes as we had predicted. Second, the findings for sex frequency might reflect gender differences in how sexual activity is factored into fairness assessments. For example, men might be more likely to view the frequency of sexual activity as a relationship outcome that should be factored into fairness evaluations. Our speculations here point to a number of intriguing avenues for future research.

### Limitations

Although our study has helped link equity, gender, and household arrangements, several limitations must be noted. First, our use of two global measures of relationship quality (relationship satisfaction and sex frequency) could be improved upon with multidimensional metrics. Additionally, we do not have information on partners’ employment status so we are unable to ascertain whether the respondents were in dual-earner or single-earner couples.

Our question about housework does not address that household chores and childcare may be perceived as separate issues. Therefore, we were unable to discriminate between perceived fairness in the division of childcare work and perceived fairness in the division of household chores. In this study, focusing on childcare as separate from housework would entail limiting our sample to only those married/cohabitors with children, losing 38 percent of our sample. While some researchers have excluded childcare from their conceptualization of housework [5], others have explicitly addressed patterns of caring activities [6]. However, our focus was on the perceived equity of the division of housework rather than direct contributions to specific tasks.

Another limitation is that we measured individuals’ perceived fairness for their partner rather than focusing exclusively on fairness for oneself. Thus, the measurement of fairness for others could reflect biased reports. For example, we did not ask what the respondents thought their partners might perceive as fair or not. Both scenarios would have presented an opportunity to demonstrate arguments *against* perceived equity, although arguably weak ones.

Our assessments of perceived unfairness to partner were more in line with the equity framework—individuals’ perceptions of fairness are reliant on their own input relative to what they perceive is the input of others, which is, in itself, a subjective assessment. These subjective assessments of perceived fairness might be subject to fewer estimation problems than self-report measures of actual housework time for oneself or one’s partner. Nevertheless, future research adopting time diaries (e.g., [21]) and actor-partner interdependence models would provide a more rigorous methodology and statistical approach to a question that very much hinges on partner interactions and conversations.

These correlational data preclude our ability to make causal statements about the relationship between equity evaluations and relationship quality. For example, relationship quality might affect individuals’ reports of perceived fairness. This problem with endogeneity points up some important avenues of study for future researchers. Given that much has changed in the national conversation about relationships and the gendered division of labor since our data were collected in 2008, we further hope that researchers will replicate these results with more recent data.

Although our sample was large, it was not probability-based. As is true of Internet samples in general, our collection of data from an Internet website yielded a non-representative convenience sample of respondents with relatively higher household incomes and educational levels when compared to the national population [73, 74]. However, large-scale Internet surveys tend to be more diverse in age, socioeconomic status, and geographic region than non-probability samples generated by many traditional data-gathering methods [75]. Ultimately, since this sample is not a population representative sample, there may be some inconsistencies in our results compared to the broader literature. Yet, our results help point to issues that would benefit from further research.

It is unclear how the sampling procedure used in this study would produce different associations among these variables compared to other recruitment methods. We also note a very important difference between our sample and the ones often solicited on websites. Many Internet samples are taken from specialty websites that draw in visitors who share particular demographic factors or interests. We had access to a major and multifaceted website that draws visitors for diverse reasons including hard news, trending popular culture news, weather, sports, and financial updates. Enabling more robust comparisons between groups, our broad access yielded a sample that was more diverse with respect to gender, age, socioeconomic status and geographic region than most Internet surveys; in fact, our sample was much more diverse than non-probability samples generated by many of the more traditional data-gathering methods [75].

### Conclusion

Given the multidimensional nature of relationships, we have provided some insight into how fairness is perceived across different relationship domains and how this varies based on the person for whom unfairness is perceived (self or other). Moreover, there is support for concluding that a gendered lens of equity evaluation continues to provide a basis for acknowledging some of the evaluative standards that couples use in their relationships.

## Supporting information

S1 Dataset (CSV)
